# The association between serum ß-hydroxybutyrate and milk fatty acid profile with special emphasis on conjugated linoleic acid in postpartum Holstein cows

**DOI:** 10.1186/s12917-016-0679-7

**Published:** 2016-03-11

**Authors:** Pedro Melendez, Pablo Pinedo, José Bastias, Maria Paz Marin, Carolina Rios, Consuelo Bustamante, Natalia Adaro, Mario Duchens

**Affiliations:** College of Veterinary Medicine, University of Missouri, Columbia, MO 65211 USA; Texas A&M AgriLife Research & Extension Center, College of Veterinary Medicine and Biomedical Sciences, Texas A&M University System, Amarillo, TX 79106-1769 USA; College of Veterinary Medicine, University Santo Tomas, Viña del Mar, Chile; College of Veterinary Medicine, University of Chile, Santiago, Chile; Present Address: Department of Animal Sciences, Colorado State University, Fort Collins, CO 80523 USA

**Keywords:** Ketone bodies, Conjugated linoleic acid, ß- hydroxybutyrate

## Abstract

**Background:**

Ketogenesis is a secondary metabolic pathway to provide energy to dairy cows during early lactation; however when the production of ketone bodies (acetoacetate, acetone, ß- hydroxybutyrate) is above certain levels a subclinical disorder may appear. The aim of the present study was to investigate the association between serum concentrations of ß- hydroxybutyrate (BHBA) and fatty acid (FA) profile of milk with emphasis in conjugated linoleic acid (CLA) in a population of early lactation Holstein cows. Fifty cows between parity 1 and 5, ranging from 14 to 21 days in milk, were randomly selected from 3 farms of the central area of Chile for determination of serum BHBA concentrations, milk fat content, and milk FA profiles.

**Results:**

Cows were divided in low (*n* = 26) and high (*n* = 24) BHBA groups considering the median value of the serum concentration of BHBA (0.7 mmol/L) (SEM = 0.094). Mean milk fat % was 3.45 % and 3.60 % for cows in the low and high BHBA groups, respectively (*P* = 0.15). Concentrations of several FA were significantly different between both groups. Specifically, mean CLA concentrations were 0.40 % (4 ± 0.03 g/kg) and 0.33 % (3.3 ± 0.03 g/kg) for the low and high BHBA groups, respectively (*P* = 0.05).

**Conclusions:**

It is concluded that early postpartum cows with serum BHBA > 0.7 mmol/L tended to have higher milk fat % and had significantly lower concentrations of CLA than early postpartum cows with BHBA ≤ 0.7 mmol/L.

## Background

Ketogenesis is a common metabolic pathway to provide energy to dairy cows during early lactation; however the synthesis of large amounts of ketone bodies (acetoacetate, acetone, and beta hydroxyl butyrate [BHBA]) may result in a typical metabolic disorder denominated ketosis [4]. Several studies have shown that high levels of BHBA are associated with increased risk of periparturient diseases, losses in milk yield and impaired fertility [4, 9, 11, 12]. In addition, increased concentrations of BHBA were associated with greater milk fat and lower milk protein percentages in the first Dairy Herd Improvement test day of lactation [4].

Conjugated linoleic acid (CLA) is one component of milk fat that might be beneficial to human health due to anticarcinogenic properties [15]. However, this potential benefit has been described for only few isomers of CLA, especially for those called CLA cis-9, tans-11. Strategies such as commercial sources of CLA added into dairy cattle diets is one potential means of increasing CLA content of milk [5]. Conversely, there might be several factors decreasing the levels of CLA in milk such as feeding excessive grains (starch), corn silage and products rich in polyunsaturated fatty acids (e.g. vegetable oils) [8]. As stated previously, the association of high levels of ketone bodies and greater milk fat percentage in dairy cattle has been reported [3, 4, 10]. However, these studies did not evaluate fatty acid (FA) profile in milk. The hypothesis of this study was that there is a relationship between BHBA levels and FA profile of milk in early lactation Holstein cows. The objective of this study was to determine the association of plasma concentrations of BHBA and FA profile of milk with special emphasis in CLA in early lactation Holstein cows.

## Results and discussion

The median value of BHBA concentration was 0.7 ± 0.094 mmol/L. Twenty six cows were classified as having a plasma concentration of BHBA of ≤ a 0,7 mmol/L and 24 cows as having a plasma concentration of BHBA of > 0,7 mmol/L. The reason for using the median value as cut-off point was to develop two groups with comparable number of animals. When the cut-off for subclinical ketosis of 1.2 mmol/L of BHBA [14] was considered, only 7 cows (14 %) were above this concentration, and 43 cows were below this value; consequently there was not sufficient statistical power to conduct the analysis.

For all study variables (fat % and fatty acids) the interaction of group by parity was not significant; therefore only main effects are reported. The milk fat content (g/kg) of cows with high and low BHBA was 3.60 % and 3.45 %, respectively (*P* = 0.15). Consequently, there was a tendency for milk fat content to be higher in the group of cows with BHBA > 0.7 mmol/L. This is in agreement with the study conducted by Duffield et al [4] where cows with higher levels of BHBA had higher concentration of milk fat than cows with low levels of BHBA. This association might be reasonable since BHBA is used as precursor for milk fat synthesis in the mammary gland. Short- and medium-chain FA (4 to 14 carbons) and a portion of the 16-carbon FA are derived from de novo synthesis from acetate and to a lesser extent butyrate [1]. Conversely, preformed FA that account for the remaining 16-carbon and all of the longer-chain FA (>16 carbons) are taken up from the circulating plasma pool. These long FA originate from absorption from the digestive tract or mobilization from body reserves. Adipose tissue mobilization accounts for less than 10 % of preformed FA in milk fat, except during periods of negative energy balance when their proportion increases substantially [1].

In Table 1, the least squared mean concentration and SEM of milk FA (g/kg of fat) of both group is presented. An interesting finding of this study was that the concentration of CLA in milk in cows with BHBA ≤ 0.7 mmol/L was higher than in cows with levels of BHBA > 0.7 mmol/L (*P* ≤ 0.05). Differences were also found for 18:1 (all isomers). In addition, results from the multivariate regression model (Table 2) show there was a tendency that for each 0.1 mmol/L of BHBA in plasma, CLA (all isomers) decreased in 0.2 g/kg of total fat (*P* = 0.15). This association was established correcting for milk yield, solid contents, somatic cell counts and milk urea nitrogen. The total r ^2^ for the predicting equation was 0.59, indicating that 59 % of the variation of CLA in milk was explained by the current model. The partial slope for BHBA was essentially linear, without evidence of polynomial effects. Fatty acid metabolism in the rumen has a major influence on the FA profile in milk [7]. The CLA are present at higher concentrations in ruminant products because they are formed in the rumen from dietary linoleic and linolenic acid. This transformation is due to via three major processes carried out by rumen microbes: lipolysis, biohydrogenation and isomerization. These mechanisms are dependent on the type and amount of fat entering the rumen [2] and ruminal pH [14]. The question arising is why levels of blood BHBA > 0.7 mmol/L might be negatively related to CLA in milk. BHBA is synthesized in the liver through the process of ketogenesis [6] and in the rumen through butyrate-producing bacteria [7]. In addition, all bacteria that produced substantial quantities of *cis*-9, *trans*-11-CLA, *trans*-11-18:1, or both from linoleic acid were butyrate producers (mostly *Butyrivibrio fibrisolvens*). However, not all butyrate producers yield *cis*-9, *trans*-11-CLA or *trans*-11-18:1. Other source of CLA is cell membrane from protozoa. Protozoa contained at least 2 to 3 times more unsaturated FA than bacteria. These unsaturated FA included CLA and *trans*-11-18:1, which were more than 8- and 3-fold more abundant in protozoa than in bacteria, respectively. As a result, the flow of FA from protozoa accounted for between 30 and 43 % of the CLA and 40 % of the *trans*-11-18:1 reaching the duodenum [17]. The contribution of protozoa to the flows of 16:0 and 18:0 to the duodenum was less than 20 and 10 %, respectively. Although protozoa do not themselves produce CLA and *trans*-11-18:1 by their own metabolism; they might be expected to have a significant influence on CLA and *trans*-11-18:1 available to the host animal [17]. In this sense, the content of CLA in milk is explained by several highly complex mechanisms. Since butyrate is precursor for short and medium chain milk FA it is reasonable to suggest that higher concentrations of blood butyrate would increase milk fat through fatty acids < 16 C; and would decrease proportionally long chain fatty acids. This is partially true, because in the present study most of short and medium chain FA, and 18:1 were higher in cows with BHBA > 0.7 mmol/L than in cows with BHBA ≤ 0.7 mmol/L. Accordingly, even though, *trans-11* C18:1 and *cis-9, trans-11* CLA are the predominant trans FA intermediates produced from the ruminal metabolism of linoleic acid; ruminal biohydrogenation pathways are dynamic, allowing the production of a wide range of positional and geometric isomers as well as modified FA such as hydroxy and keto derivatives [7, 13].

**Table 1:** Least squared mean concentration and SEM of milk FA in cows with BHBA plasma concentrations ≤ 0,7 mmol/L (*n* = 26) and > 0,7 mmol/L (*n* = 24)^a^

Fatty acid (%)	BHB ≤ 0,7 mmol/L	BHB > 0,7 mmol/L	SEM	*p*-value
4:0	2.43	2.35	0.1	0.63
6:0	1.75	1.13	0.1	0.0007
8:0	1.77	0.77	0.32	0.0001
10:0	0.95	0.4	0.35	0.0001
12:0	2.09	1.29	0.13	0.001
14:0	6.23	7.87	0.32	0.003
14:1	0.3	0.21	0.04	0.66
15:0	0.78	0.63	0.03	0.01
16:0	28.68	28.67	0.5	0.92
16:1	1.12	1.68	0.08	0.0001
17:0	0.82	0.96	0.03	0.0008
18:0	17.96	18.25	0.56	0.42
18:1 (all isomers)	30.69	32.67	0.43	0.04
18:2 (all isomers CLA)	0.4	0.33	0.03	0.048
18:2 (all isomers no CLA)	0.52	0.43	0.04	0.28
20:0	0.51	0.47	0.03	0.43

^a^All models included the effect of parity (primiparous, multiparous) and farm (1, 2, and 3); consequently reported least squared means for all fatty acids are adjusted for these two variables. For all FA, the interaction between group and parity was not significant

**Table 2:** Multivariate regression model for CLA (g/kg) in milk. Coefficient of determination (*r* ^*2*^) = 0.594

Independent variables	Coefficients	SEM	*P*-value
Constant	6.68	4.76	0.06
BHBA (per 0.1 mmol/L)	−0.22	0.14	0.15
Fat (%)	−0.34	0.16	0.05
Protein (%)	−0.56	0.98	0.58
Milk urea nitrogen (per 1 mg/dl)	0.30	0.18	0.14
Somatic cells count (per 1,000 cells/ml)	−0.00024	0.0001	0.05
Average milk yield up to 60 days in milk (per 1 kg/day)	0.0041	0.048	0.25

Finally, if the negative association between levels of BHBA in blood and proportion of CLA in milk remain consistent in further studies, especially in cows with high levels of BHBA (>1.2 mmol/L), it would be another reason to consider essential the prevention of high levels of ketone bodies, if the dairy industry is looking for milk and its derivatives with high levels of CLA.

## Conclusions

Early postpartum cows with plasma BHBA > 0.7 mmol/L tended to have higher milk fat content and significantly lower proportion of CLA than early postpartum cows with BHBA ≤ 0.7 mmol/L. For each 0.1 mmol/L of BHBA in plasma, CLA (all isomers) decreased in 0.2 g/kg of total fat.

## Methods

### Dairy farms

The study was conducted during 2010 in three dairy farms from the central area of Chile. The study was performed in accordance with the recommendations of the University Santo Tomas animal care guidelines. No procedure causing more than momentary discomfort was performed during this study. In addition, all the animal related actions had the consent of the University Animal Care Committee and the dairy owners. Average herd size was 350 lactating Holstein cows. Rolling herd average milk yield was 10,000 to 12,000 kg ME 305 and selected farms followed similar management practices. Farms housed cows in sand-bedded free-stalls and milked 3 times a day. Feed management consisted of a unique total mixed ration based on alfalfa hay, corn silage, corn grain, soybean meal, wet brewer grain, wheat bran, distiller derivative grain solubles, minerals and vitamins. Farms used computerized record systems; consequently individual milk yield was recorded daily based on computerized milking parlors. During early lactation cows were subjected to a postpartum health management protocol. After calving cows were checked for retained fetal membranes and the development of milk fever. Daily clinical examination and rectal temperature were conducted until 14 days postpartum. Milk BHBA colorimetric testing (Ketotest, Nagoya, Japan) was conducted at once between 14 to 21 days postpartum. Sick cows were treated as needed.

### Study design

In order to find a difference in 0.1 ± 0.1 g of CLA per kg of milk fat, with a 95 % of confidence and 80 % of power, a sample size of 13 cows per group was required. Weekly visits for the 3 farms were conducted between May and August of 2010 until 50 cows within 14 and 21 days in milk were obtained. Blood and milk samples were collected from each cow the same day of assortment. Blood sample for plasma collection was obtained from the coccygeal vessels using a vacuatainer system and centrifuged at 4000 × g for 10 min. Plasma was separated and stored in plastic tubes and frozen at - 20 °C until analysis. Plasma samples were submitted to the laboratory of clinical pathology of University of Santo Tomas, Viña del Mar, Chile. The determination of plasma BHBA concentrations was conducted using a commercial kit (Pointe Scientific, Inc. BHA Set., Lincoln Park, MI, USA), based on an enzymatic-colorimetric method [16]. Samples were assayed in duplicate with a coefficient of variation of 4.8 %.

A milk composite sample was obtained during the morning milking in a plastic tube with preservative (2-bromo-2-nitropropane- 1-2-diol).and immediately after collection submitted to the laboratory of biochemistry at the Catholic University of Valparaiso, Chile, for milk fat content and milk FA profile analysis. The determination of milk fat (g/kg) was determined using infrared spectroscopy (MilkoScan™ Minor, DK-3400, Hilleroed, Denmark). Milk samples were analyzed for individual FA by Gas Chromatograph-Mass Spectrometry (GCMS) of butyl esters. The identification of fatty acids was based on the comparison of retention times of standard methyl esters ranging from C4 to C24 (Sigma-Aldrich, St. Louis, MO, USA). Calibration standards were achieved from seven different concentrations, depending on the particular FA. Quality control samples were prepared at four different concentrations.

The methodology consisted as follow: 0.5 ml of milk was placed into test tubes with teflon-lined screw caps, followed by the addition of 750 *μ*l of n-butanol. Samples were vortexed at low speed while 75 *μ*l of acetyl chloride was added. Then, samples were gassed with N_2_, capped tightly, and heated at 100 °C for 1.5 h. After samples cooled to room temperature, 5 ml of 6 % K_2_CO_3_ and 1 ml of hexane were added, and the samples were vortexed for 30 s. Samples were centrifuged for 20 min at 2500 *g*, and the bottom layer was aspirated and discarded. The remaining layer was washed four times (20 min at 2500 *g*) with distilled, deionized water. The upper layer was removed and placed in injection vials for analysis. Fatty acid analysis of butyl esters was conducted with a GCMS model QP5050A, series Splitless (Shimadzu®, Shimadzu Europa GmbH, Albert-Hahn-Str. 6-10 47269, Duisburg, Germany).

Results were expressed as percentage relative to each FA detected in the chromatogram according to its retention time and identification based on pure standard. Subsequently, the median value for BHBA concentrations (0.7 mmol/L) was considered as a cut-off value to obtain balanced numbers of animals in the low and high BHBA groups. Milk fat (g/kg) and FA (g/kg) content between the 2 groups were analyzed by ANOVA, developing a mixed model that also included parity (primiparous, multiparous) and farm (1, 2, 3). Potential interaction between group and parity was also explored. Least squared means were estimated and tested for significant differences. In addition, a multivariate regression model was conducted to predict total CLA (g/kg) in milk from plasma BHBA as a continuous variable, correcting for fat and protein % in milk, milk urea nitrogen (mg/dl), somatic cell count (cells/ml) and average milk yield until 60 days in milk (kg/d). Statistical analysis was performed using the corresponding procedure of SAS (release 9.0; SAS Institute Inc., Cary, NC). Statistical significance was set at *P* ≤ 0.05; a tendency was set at 0.05 < *P* ≤ 0.15.
